# Dietary Effect of *Brevibacillus laterosporus* S62-9 on Chicken Meat Quality, Amino Acid Profile, and Volatile Compounds

**DOI:** 10.3390/foods12020288

**Published:** 2023-01-08

**Authors:** Xiangfei Liu, Aijin Ma, Tongxin Zhi, Dan Hong, Zhou Chen, Siting Li, Yingmin Jia

**Affiliations:** Beijing Advanced Innovation Center for Food Nutrition and Human Health, School of Food and Health, Beijing Technology and Business University, Beijing 100048, China

**Keywords:** meat quality, amino acid, volatile compounds, microbial agent

## Abstract

Probiotics are being used in diets to improve the quality of chicken meat. The aim of the study was to investigate the effects of dietary supplementation with *Brevibacillus laterosporus* S62-9 microbial agent on the meat quality, amino acids, and volatile compounds of chicken. The experiment was carried out with 160 1-day-old Arbor Acres male broiler chickens, rearing for 42 d. The chickens were randomly divided into two groups of 8 replicates each, with 10 chickens in each group. No supplement was added to the basal diet in the control group and *Brevibacillus laterosporus* S62-9 microbial agent was added to the diet of the experimental group. At the end of the experiment, the meat quality, meat chemical composition, amino acid composition, and volatile compounds of chicken were determined. The results showed that pH (*p* < 0.05), pressing loss (*p* < 0.05), cooking loss (*p* < 0.05), and shear force (*p* < 0.01) were notably decreased, the percentage of breast meat (*p* < 0.01), protein content (*p* < 0.05) were visibly increased, and remarkable changes were observed in the amino acid composition (change in seven amino acids) and volatile compounds profile (an increase of about 20-fold in the contents of 1-octen-3-ol and hexanal). In summary, it was found that *Brevibacillus laterosporus* S62-9 microbial agent can be used as a novel and effective feed supplement to improve the nutritional quality and flavor characteristics of broilers.

## 1. Introduction

For a long time, antibiotics have been widely used in broiler farming in order to prevent disease and promote growth [1,2]. However, the indiscriminate use of antibiotics in broiler farming has led to the production of drug-resistant microorganisms, which could spread to humans by consuming such chicken meat. The increased incidence of pathogenic bacteria that are resistant to many different types of antibiotics and cause concern for animal and human health has made this resistance a serious public health issue [3]. The use of antibiotics has been restricted to varying degrees given the public health [4,5,6,7], which has led to the emergence of antibiotic alternatives such as feed acidifiers, antibodies, phages, antimicrobial peptides, prebiotics, and probiotics, the most intriguing of which are bacteriocin-producing probiotics [8]. Meanwhile, probiotics have been proven to promote growth performance [9,10] and meat quality [11] in broilers by maintaining the microbial balance and protecting intestinal health [12,13]. In the study of Mohammed et al. [14], it is indicated that the probiotic supplement could enhance bone mass and meat quality to improve broiler welfare and production. Moreover, a recent study has determined that broilers were given an enhanced immune and antioxidant capacity by dietary supplementation of compound probiotics, while antibiotics lacked such merits [15]. The survival of probiotics is threatened by the unfavorable environment in the gastrointestinal tract such as acid (the pH can reach as low as 1.5 in the stomach) and bile (the bile concentrations encountered in the intestine) [16]. Due to its pH and bile salt resistance in the gastrointestinal tract, as well as its metabolite expression that can improve the overall condition of birds, the poultry industry is moving toward the use of *Bacillus*-based probiotic products [17].

The application of probiotics in poultry is not only limited to growth performance, gut health, and body immunity but also has a key role in improving meat quality such as water-holding capacity, tenderness, lipid oxidation stability, sensory characteristics, and microbial safety in broilers [18]. Along with the improvement in tenderness, it was reported by Liu et al. [19] that the use of probiotic supplementation has the potential to improve the meat quality of broilers by increasing the flavor and nutritional substances. In contrast to the application of probiotics in fermented meat products [20,21], the mechanisms associated with the improvement of meat quality by adding probiotics to the diet are not clear [22]. The mechanism may be related to the involvement of probiotics in regulating muscle chemical composition and flavor substance deposition by maintaining intestinal balance [15] and enhancing nutrient absorption [23].

With the prevalence of broiler farming and the increased demand for meat quality, the use of probiotics instead of antibiotics to improve meat quality and maintain food safety is becoming a hot field. Antimicrobial capacity is critical in biocontrol [24]. Similarly, organisms that can produce a product that will inhibit the growth or kill existing organisms in the intestinal milieu have a distinct advantage. Previous studies have shown that *Brevibacillus laterosporus* S62-9 exhibits a wide range of antimicrobial activity against fungi and bacteria. Meanwhile, good thermal tolerance, and good acid and bile salt resistance were found in *Brevibacillus laterosporus* S62-9 in the previous study. Thus, the *Brevibacillus laterosporus* S62-9 microbial agent was found to be a promising microbial agent for broiler feeding and might have favorable effects on the nutritional quality and flavor characteristics of meat. Furthermore, the effect of the probiotic, *Brevibacillus laterosporus* S62-9, on related meat quality in broilers has not been investigated previously. This study was designed to evaluate the effects of dietary supplementation with *Brevibacillus laterosporus* S62-9 on nutritional quality and flavor characteristics of chicken meat by investigating growth performance, carcass traits, meat quality, amino acids, and volatile compounds in broiler chickens.

## 2. Materials and Methods

### 2.1. Microbial Agent Preparation

In the previous study by our group, *Brevibacillus laterosporus* S62-9 was resistant to heat, acid, and bile salts in vitro. Strain S62-9 had no observable resistance to 10 types of antibiotics tested and was thus considered to be a biosafe strain. *Brevibacillus laterosporus* S62-9 was scraped from the agarslantculture medium of the strain stored at 4 °C to the nutrient broth medium for activation (37 °C, 240 rpm/min, with constant temperature and shaking for 24 h), fermenting in a specific high-yielding fermentation medium for 48 h (37 °C, 240 rpm/min), and by spray drying into powder to get a microbial agent with the main component *Brevibacillus laterosporus* S62-9 up to 10^9^ cfu/g.

### 2.2. Animals and Diets

The procedures and experiments of animals were approved by the Experimental Animal Welfare and Ethics of the Institute of Animal Science, Chinese Academy of Agricultural Sciences (ethical approval code IAS-2022-135). This experiment was conducted at the Changping Experimental Base of the Beijing Institute of Animal Husbandry and Veterinary Medicine, Chinese Academy of Agricultural Sciences, which was carried out with 1-day-old Arbor Acres male broilers, divided into 2 homogeneous experimental groups (control group = CN; *Brevibacillus laterosporus* S62-9 = BS, diet containing probiotic at 10^6^ cfu/g), each group contained 80 broilers and there were no significant differences in initial weight between the two groups. The diet composition and nutrition were according to GB/T 5916 2020 (Table 1).

### 2.3. Slaughter and Sample Collection

On day 42 of the feeding experiment, 10 broilers (1 bird/replicate) were randomly selected from each treatment for sample collection. All selected birds were weighed respectively and slaughtered by cutting the jugular vein after being stunned by carbon dioxide in a professional slaughtering room. According to a standardized procedure (NY/T 8232004) to determine the dressing percentage (%), semi-eviscerated percentage (%), eviscerated percentage (%), abdominal fat (%), breast muscle (%), and thigh muscle (%). Dressed weight was recorded after defeathering and exsanguination. The dressing percentage (%) was calculated as:

(1) $$\mathrm{dressing}\;\mathrm{percentage}\;\left(\%\right)=\times 100$$

After removing trachea, esophagus, crop, intestine, spleen, pancreas, gallbladder, gastric contents, and corneum membrane, the semi-eviscerated weight was recorded. The semi-eviscerated percentage (%) was calculated as:

(2) $$\mathrm{semi}-\mathrm{eviscerated}\;\mathrm{percentage}\;\left(\%\right)=\times 100$$

Finally, the heart, liver, proventriculus, gizzard, lung, and abdominal fat were removed, and the eviscerated weight was recorded. The eviscerated percentage (%) was calculated as:

(3) $$\mathrm{eviscerated}\;\mathrm{percentage}\;\left(\%\right)=\times 100$$

The abdominal fat (%) was calculated as:

(4) $$\mathrm{abdominal}\;\mathrm{fat}\;\left(\%\right)=\times 100$$

The breast muscle (%) was calculated as:

(5) $$\mathrm{breast}\;\mathrm{muscle}\;\left(\%\right)=\times 100$$

The thigh muscle (%) was calculated as:

(6) $$\mathrm{thigh}\;\mathrm{muscle}\;\left(\%\right)=\times 100$$

After cutting the carcasses, the breast and thigh meat (10 birds/group) were selected for determination of pH, meat color, pressing loss, cooking loss, and shear force, and the remaining chicken meat was stored at −20 °C for subsequent analysis.

### 2.4. Meat Quality Assessment

The pH of breast and thigh muscle was measured at 45 min and 24 h (stored in air at 4 °C for 24 h) after slaughter using a pH meter (HI99163, HANNA, Woonsocket, USA) 1 cm deep into the center of the meat tissue, and the right breast meat (near the bone side) and thigh meat color was measured at 24 h (stored in air at 4 °C for 24 h) after slaughter by a colorimeter (CR-400, KONICA MINOLTA, Tokyo, Japan). Specifically, at the time of determination, the surface color of the breast muscle close to the bony side was determined, and three positions from the top to the bottom were determined and averaged. Three values were measured in duplicate at the surface of different thicknesses of the same thigh meat, then the average value was calculated as the thigh meat color determination value; the pressing loss and cooking loss were used to assess the water-holding capacity of the chicken. Pressing loss was measured through the differences in weight before and after pressurization [25], and the pressing loss was calculated as:

(7) $$\mathrm{pressing}\;\mathrm{loss}\;\left(\%\right)=\times 100$$

The operations were as follows, wrapping in gauze after weighing (volume 1.5 × 1.5 × 1.0 cm), and then wrapped in 18 layers of quantitative filter paper, placed on a YYW-2 strain-controlled lateral limitless pressure gauge and pressurized to 15 kg for 5 min, then removed and weighed; cooking loss was measured according to Liu [26]. The cooking loss was measured through the differences in weight before and after cooking, and the cooking loss was calculated as:

(8) $$\mathrm{cooking}\;\mathrm{loss}\;\left(\%\right)=\times 100$$

Briefly, after samples were cut in the direction of the muscle fibers (volume 6 × 3 × 3 cm), weighed accurately, then placed in a steaming bag, and heated at 90 °C in a water bath until the central temperature of the meat sample reached 75 °C. The measurements of shear force were conducted as described by Wang [27] with appropriate modification. After the meat samples had cooled to room temperature, the moisture of the surface was blotted up by using filter paper. The meat samples were then cooled to 4 °C of central temperature and drilled in a direction which was parallel with the muscle fibers using a circular sampler of 1.27 cm in diameter, with holes in the length of 3 cm, at least 5 cm away from the edge of the meat sample and 6 cm between the edges of the two sampling locations, excluding those with obvious defects, and selected 3 samples using a texture analyzer (TA.XT.Plus, SMS) to determine the shear force. 

### 2.5. Chemical Composition and Amino Acid Composition Analysis

Moisture, protein, fat, and ash were determined with references to GB5009.3-2016, GB5009.6-2016, GB5009.5-2016, and GB5009.4-2016, respectively.

The amino acid (AA) composition of the breast and thigh muscles was determined according to GB5009.124-2016. Basically, the meat samples were hydrolyzed in a hydrolysis tube at a constant temperature of 110 °C in 16 mL HCl (6 mol/L) for 22 h. The hydrolysis products were transferred and diluted in a 50 mL volumetric flask using a fixed volume of 0.02 mol/L HCl. After mixing, 1 mL of the solution was transferred to an evaporation flask and rotary evaporated to dry at 55 °C in a water bath, then dissolved with ultrapure water, evaporated with spinning, and repeated the above two steps twice. After this, the amino acids were dissolved in 2 mL of HCl (0.02 mol/L), filtered (0.22 μm), and determined using a fully automated amino acid analyzer (LA8080, Hitachi, Tokyo, Japan), by comparing with standards (013-08391, Wako, Tokyo, Japan) to determine individual amino acids.

### 2.6. Volatile Profile Analysis

According to Ho [28] with slight modification, headspace solid-phase microextraction (HS-SPME) was used for the analysis of volatile components. The meat was transferred to a 20 mL glass headspace vial after weighed 5.0 ± 0.002 g, 1 µL of 2-methyl-3-heptanone was added as an internal standard, sealed immediately, and placed on an SPME fully automated loading system (PAL RSI, Zwingen, Switzerland) to extract the aroma material at 60 °C, equilibrated for 20 min and extracted for 40 min. Gas Chromatography-Mass Spectrometry (GC-MS) was performed using a gas chromatograph-mass spectrometer (GC-MS D7890-5977B, Agilent, Palo Alto, USA) with a DB-WAX column (30 m × 0.53 mm, 1 μm); helium as a carrier gas, flow rate 1.2 mL/min; non-split mode; inlet temperature 250 °C; determination procedure: initial temperature 40 °C, hold for 3 min, and ramp up at 5 °C/min to the mass spectrometry electron energy was 70 eV; the electron ionization source; the transmission line, ion source, and quadrupole were 280 °C, 230 °C, and 150 °C, respectively; and the mass scan range was 55–500 *m*/*z*. The volatile compounds were qualitatively determined based on the NIST4 mass spectrometry library, RI, and the descriptions of standard odor, and the area normalization method was adopted to the content of volatile compounds. ROAVs were calculated based on the volatile compounds content and threshold values in water.

### 2.7. Sensory Analysis

The evaluation of sensory was performed according to Bosque [29] with minor modifications. The 9-point evaluation form was adopted for the cooked meat evaluation of the breast and thigh muscle separately in the sensory evaluation experiment. The meat samples used for sensory analysis were thawed overnight at 4 °C the day before the evaluation. The samples, after finishing thawed samples, were placed in steaming bags and cooked in water at 80 °C before the central temperature reached 75 °C, then placed in three trays with coded digits. The meat samples were to be evaluated by nine experts who had been trained three times before. The sensory evaluation involved nine panelists (4 females and 5 males, aged 24–42), who were invited from the Institute of Animal Science, Chinese Academy of Agricultural Sciences, with professional training and rich experience in sensory evaluation of meat-related products. 

### 2.8. Statistical Analysis

The analysis of data was performed with an independent sample *t*-test using SPSS 24.0 programs (SPSS, Inc., Chicago, IL, USA). Results are expressed as the mean ± standard deviation (SD). Statistical significance was indicated by *p* < 0.05.

## 3. Results

### 3.1. Growth Performance and Carcass Traits

It was shown in Table 2 and Table 3 that the addition of *Brevibacillus laterosporus* S62-9 microbial agent to the diet affected the growth performance and carcass traits of broilers. Compared to the control group, after the addition of *Brevibacillus laterosporus* S62-9 to the diet, the feed conversion ratio (FCR) was reduced, accompanied by average daily gain (ADG) of 22–42 days, body weight (BW) of 42 days and breast muscle yield was increased. There were no significant changes in carcass weight, dressing percentage, semi-eviscerated percentage, eviscerated percentage, and the percentage of abdominal fat and thigh muscle in the experimental group.

### 3.2. Meat Quality

As shown in Table 4, the addition of *Brevibacillus laterosporus* S62-9 microbial agent to the diet significantly reduced brightness (L*) shear and increased redness (a*) of breast meat, while 45 min, 24 h pH, pressing loss, cooking loss, and shear force of thigh meat was decreased.

### 3.3. Chemical Composition and Amino Acid Composition Analysis

Table 5 shows that dietary supplementation with the *Brevibacillus laterosporus* S62-9 microbial agent affected the chemical composition (moisture, crude protein, crude fat, and crude ash) of broiler meat. It is worth noting that the protein content of the breast muscle and the fat content in the thigh meat of broilers in the experimental group were dramatically higher than those in the control group.

The AA content of breast and thigh muscles is given in Table 6. In the breast muscles, serine, threonine, alanine, methionine, phenylalanine, arginine, and total essential amino acids were higher than those in the control group. Moreover, the content of total AA was increased by the addition of *Brevibacillus laterosporus* S62-9 microbial agent. There was no significant difference between the experimental and the control group in the thigh muscle, but there was a tendency to increase the content of AA in the experimental group.

### 3.4. Volatile Compounds of Meat

Table 7 presents the variety and content of volatile compounds in breast and thigh muscles. Thirty-one volatile compounds were identified in the breast and thigh, which were classified into aldehydes, alcohols, acids, and others in accordance with their functional groups. There was a large variation in aldehydes and alcohols between the control group and the treatment group. On basis of the ROAVs (Table 8), four major components that varied widely and contributed greatly to the volatile compounds can be identified as 1-octen-3-ol, hexanal, octanal, and nonanal.

### 3.5. Sensory Analysis

The effects of the addition of *Brevibacillus laterosporus* S62-9 microbial agent to the diet on the sensory properties of cooked meat are shown in Table 9. In the cooked breast meat, the treatment obviously enhanced tenderness and juiciness. In the cooked thigh meat, the experimental group got higher scores for aroma and tenderness than those in the control group.

## 4. Discussion

Antibiotic growth promotions (AGPs), as feed additives, were used to enhance and improve growth performance and carcass traits of broilers in the past [30,31]. However, as consumer demand for healthier, safer, and tastier meat increases, feeding microorganisms are becoming a suitable alternative to antibiotics for improving the broiler gut microbiota and enhancing broiler immunity, thereby improving meat quality and promoting broiler growth [32,33,34]. This experiment was conducted to study the effects of the *Brevibacillus laterosporus* S62-9 microbial agent in the diet on the growth performance, carcass traits, meat quality, and flavor of broilers.

In this study, the addition of *Brevibacillus laterosporus* S62-9 microbial agent to the diet significantly reduced FCR and increased BW at 6 weeks of age but did not significantly alter ADFI. In the present study improved BW and FCR of birds might be justified by the fact that taking probiotics increases the birds’ ability to utilize nutrients. This result was in line with the findings of Wang [35] that the addition of *Bacillus laterosporus* KC1 to the diet significantly increased broiler weight and decreased FCR at 42 days of age. In contrast, there were some relevant studies have shown that the addition of microorganisms had no significant effects on growth performance [36]. Breast meat is considered to be the most valuable part of the chicken, and small changes in breast meat yields can have a great effect on economic returns [37]. The noteworthy changes in the percentage of breast meat of birds in the current study revealed the trend of BW of birds. Consistent with Tang et al. [38], the weight of breast meat was increased by the addition of *Bacillus subtilis* DSM 32315 to the diet, while the change in dressing percentage, semi-eviscerated percentage, eviscerated percentage, and thigh meat weight was negligible. Similar to our results, Rehman [39] reported that supplementation with probiotics had no effect on abdominal fat in broilers. It was also found that breast meat yield correlated negatively with abdominal fat [40]. In this study, the percentage of breast meat was significantly higher compared to the control group, but no adverse effects on abdominal fat deposition were found with the addition. The variations in the results about growth performance and carcass traits of broilers in response to probiotics supplementation can partly be explained by the species of probiotics, the mode of delivery, the dosage, etc.

Many studies have shown that the addition of *Bacillus* to the diet was effective in improving the quality of chicken meat. The meat quality was determined mostly by its pH, color, water-holding capacity, and tenderness [41]. The pH was regarded as a common indicator for meat quality measurement, which was a reflection of the conversion of glycogen into lactic acid in the muscle before and after slaughter [42]. Meanwhile, there was a direct relevance between pH and meat quality such as tenderness, water-holding capacity, color, juiciness, and shelf life. It was common for meat to have a pH between 5.0 and 7.0 [43]. However, depending on whether the final pH of chicken meat was too high (≥6.4), or too low (≤5.7), the meat would turn out to be either DFD (dark, firm, dry), or PSE (pale, soft, exudative) [44]. Our results suggested that the addition of *Brevibacillus laterosporus* S62-9 microbial agent to the diet could prevent these conditions and improve meat quality. Meat color was a useful indicator to evaluate meat quality. The lower L* was, the redder the meat color would be, which was the indicator of high meat quality perceived by consumers [45]. The results showed that breast muscles in the experimental group had lower L* and higher a* than the control group, which was consistent with the study by Bai [46]. This improvement in meat color will benefit meat quality and consumption. Wang [47] and Zhang [48] reported that along with lower L* in samples, the moisture content was lower, but the correlation was not found in the present study. According to Turgut [49], the variation in a* may be related to the accumulation of myoglobin and metmyoglobin in the presence of light and oxygen. However, unaffected L* and a* were reported when adding probiotics to the diet by Liu [50]. The amount of microbial additives might account for the difference. The shear force has been regarded as a basic indicator of the tenderness of the meat. Higher tenderness was associated with lower shear forces. Both the treatment groups of breast and thigh meat had lower shear force, suggesting that dietary supplements containing *Brevibacillus laterosporus* S62-9 microbial had beneficial effects on the improvement of chicken meat tenderness, especially on thigh meat tenderness, and this was strongly related to the enhancement of fat content in the thigh meat [51]. The critical role of water-holding capacity has been established. It was not only associated with juiciness [52] and tenderness [53], but also with water loss and nutrient loss. Therefore, the level of pressing loss and cooking loss reflected the quality of the meat to a certain extent. The current outcomes revealed that thigh meat had lower water loss under external pressure and heat treatment.

The present study depicted that dietary supplementation with *Brevibacillus laterosporus* S62-9 had no effect on the content of moisture and ash in both breast and thigh muscles compared to the control group. Whereas, adding *Brevibacillus laterosporus* S62-9 to the diet had different effects on protein and fat in the breast and thigh. Our study was consistent with the findings of Liu et al. [19], in which the treated group had a markedly higher protein content. The significant increase in protein content indicates that the meat quality of the chicken has been further improved, which affects further processing suitability of chicken meat. The fat content of thigh meat was significantly higher in the treated group than that in the control group, which may account for the higher tenderness and water-holding capacity of the meat [54]. In another study, no effects were observed in protein or fat content [55]. Even though the mechanism involved was unclear, it might be related to intake, digesting, and metabolic processes.

The content of amino acids in meat was closely associated with the nutritional value [56] of meat protein and meat flavor [57]. The content of essential amino acids determined the quality of the protein, while flavor amino acids contributed to the taste. The impact of feeding probiotics on the amino acid profile has been validated [38,58]. As research has shown, the amino acid content of breast meat varied significantly in the treated group, while thigh meat had a tendency to the increase in content. In the breast meat, supplementation of *Brevibacillus laterosporus* S62-9 significantly increased the content of seven amino acids, including three essential amino acids and four flavor amino acids. There were three important essential amino acids in chicken meat, threonine, methionine, and phenylalanine, which were higher in the experiment group, indicating that meat quality and nutritional value improved via the stimulative effects of *Brevibacillus laterosporus* S62-9 supplemented in the broiler diet. Flavor amino acids such as serine, alanine, glycine, and arginine can act as precursors, resulting in subtle flavors and having an important impact on meat flavor [59]. The present results led to the conclusion that the moderate dietary supplementation of *Brevibacillus laterosporus* S62-9 might improve the nutrition and flavor of chicken meat by increasing the content of essential amino acids and flavor amino acids.

The concentration of volatile compounds in fresh meat also reflects the sensory quality [60]. Volatile compound alterations via dietary means may provide a potential way to obtain better-tasting chicken for human consumption. In order to prove the effect of *Brevibacillus laterosporus* S62-9 on chicken flavor, volatile compounds were determined and none of which were identified with the distinctive meat flavor. It was clear that meat flavor was the result of the interaction of many compounds. The precursors of meat aroma substances were various types of small biological molecules which originated from the water-soluble extracts of meat. The soluble components produced meat aroma under heating, which could be summarized in three pathways: the first, by the oxidation and hydrolysis of lipids to form compounds such as aldehydes, ketones, and alcohols [61]; the second, reactions such as the Maillard reaction [62] or the decomposition and oxidation of sugars and amino acids to form volatile and non-volatile compounds; the third, reactions between the products of the two pathways above to produce numerous aroma components. As the chicken was rich in polyunsaturated fatty acids, making it easy to the oxidation of lipids [63], it was expected to be rich in volatile compounds (i.e., aldehydes, alcohols, and ketones) produced by the oxidation of lipids and fatty acids [64]. Most of the compounds were derived from linoleic acid (1-pentanol, 1-hexanol, 1-octen-3-ol, hexanal, etc.) [65] or oleic acid (1-heptanol, 1-octanol, octanal, nonanal, decanal, etc.) [66]. 

Alcohols could be produced through sugar metabolism, lipid oxidation, decarboxylation, dehydrogenation of amino acids, and reduction of aldehydes [67,68,69,70]. Unsaturated alcohols, with a lower threshold value, were considered to greatly contribute to the flavor of meat [71]. 1-octen-3-ol, which had a typical ‘mushroom’ odor [72], had a lower odor threshold compared to other alcohols [73,74] and it was detected in large quantities and there was a prominent difference between the treatment and control groups in the breast. Despite the fact that there were obvious variations in the contents of 1-pentanol in breast meat and 1-octanol and 1-heptanol in thigh meat, saturated alcohols had a higher odor threshold and were considered to have fewer effects on the meat flavor [71]. Aldehydes containing carbonyl groups are important flavor compounds originating from the oxidation of threonine, proline, lysine, and arginine in tissues [75]. Hexanal, with a ‘green/grassy’ odor [76], was detected in the highest quantities, and also there was approximately a 20-fold difference between the treatment and control groups in the breast, and hexanal had been identified as one of the most important odor-active compounds in chicken breasts [77]. Similarly, hexanal content in thigh meat was significantly increased. A relative study had shown that 1-octen-3-ol and hexanal were considered to be very important volatile compounds in chicken meat [78]. Notably, octanal, described as having “fatty/sweet” odors, also had high ROAVs in this experiment, suggesting that might contribute to the improvement of chicken flavor as important volatile compounds. Especially, the three aldehydes with high ROAV values detected in the breast of the experimental group were not detected in the control group. The result suggested that probiotic treatment might affect the flavor of breast meat by affecting the composition of volatile compounds. Acids, which generally had higher thresholds than aldehydes and alcohols, were detected at lower levels in this experiment. Although significant changes in octanoic acid and nonanoic acid contents were detected in breast meat and thigh meat, respectively, their contribution to flavor was not significant based on their ROAV values. The contents of 2-Pentylfuran in thigh meat showed a decreasing trend, and its ROAV values also showed variability, which might cause a difference in the flavor of leg meat. While the other compounds were found to contribute little to flavor based on ROAVs. In conclusion, the probiotic, *Brevibacillus laterosporus* S62-9, played a role in improving flavor characteristics through the changed composition and concentration of volatile compounds in muscle. The addition of *Brevibacillus laterosporus* S62-9 to the diet improved the flavor of chicken by mainly affecting the content of three volatile compounds, 1-octen-3-ol, hexanal, and octanal, which contribute significantly to the flavor of chicken breast meat. However, it was the combination of volatile aromas and non-volatile taste compounds that gives meat its flavor, and further analytical studies in combination with the content of free amino acid and inosine monophosphate, and gas chromatography-sniffing were required subsequently to identify compounds that may affect meat flavor and consumer preferences. PCA analyses were performed to evaluate the difference between the control group and the experimental group (Supplementary Materials). Principal coordinate axis one explained a 42.2% discrepancy across breast samples and a 43.0% discrepancy across thigh samples, respectively. The results demonstrated that dietary supplements with *Brevibacillus laterosporus* S62-9 had a significant effect on chicken quality, and this difference was more pronounced in thigh meat.

Combining sensory evaluation experiments with multiple perspectives such as texture and flavor, it could be more intuitive to evaluate the consumers’ preferences for chicken. A study by Horsted et al. [79] reported that tenderness seems to be considered a crucial consumer attribute. The results of the current study showed that the tenderness of breast and thigh meat was improved in the experimental group. Meanwhile, with the changes in the structure of chicken to be verified, not surprisingly, making it possible for consumers to favor the tenderness of both breast and thigh meat. There was evidence of strengthened flavor in the breast with the relevant physicochemical indicators although the sensory evaluation only showed a tendency to improve the flavor. The final results revealed that consumers have preferences for the chicken of the group supplemented with *Brevibacillus laterosporus* S62-9.

## 5. Conclusions

The current results suggest that the *Brevibacillus laterosporus* S62-9 microbial agent contributes to improving growth performance and breast muscle yield in broilers. Furthermore, the addition of *Brevibacillus laterosporus* S62-9 microbial agent to the diet can improve meat quality and flavor of broilers by improving pH, meat color and tenderness, increasing water-holding capacity, protein, and fat contents, and regulating the contents of amino acid and volatile compounds. In conclusion, the current findings indicate that dietary supplementation with *Brevibacillus laterosporus* S62-9 could be a useful strategy for the improvement of meat quality and flavor properties of broilers. Nevertheless, further research is needed to increase knowledge regarding the mechanisms for improving meat quality and flavor.

## Figures and Tables

**Table 1:** Ingredients and composition of the standard diets for broilers.

Ingredient	Starter Diet (%) 1–21 Days	Finisher Diet (%) 22–42 Days
Corn	51.78	52.25
Soybean meal	36.63	33.54
Wheat grain	3.02	5.02
Soybean oil	4.02	5.52
Dicalcium phosphate	1.91	1.61
Limestone	1.21	1.01
L-lysine	0.32	0.10
DL-methionine	0.19	0.10
L-threonine	0.12	0.05
NaCl	0.30	0.30
Premix ^1^	0.50	0.50
Total	100	100
Calculated nutrient level (%)		
ME (MJ/kg)	12.43	12.96
Crude protein	21.26	20.20
Calcium	1.16	0.98
Available phosphorus	0.52	0.46

^1^ Supplied per kilogram of diet: vitamin A, 8000 IU; vitamin D3, 1000 IU; vitamin E, 20.0 IU; vitamin K3, 0.80 mg; thiamine, 3.0 mg; riboflavin, 8.0 mg; vitamin B6, 5.0 mg; vitamin B12, 20 µg; pantothenic acid, 10.0 mg; nicotinic acid, 40.0 mg; folic acid, 0.6 mg; biotin, 0.2 mg; Cu (copper sulfate), 8.0 mg; Fe (ferrous sulfate), 100.0 mg; Mn (manganese sulfate), 120.0 mg; Zn (zinc sulfate), 100.0 mg; I (calcium iodate), 0.70 mg; Se (sodium selenite), 0.30 mg.

**Table 2:** Effects of dietary *Brevibacillus laterosporus* S62-9 on the meat quality of chicken meat.

Item	CN	BS	*p*-Value
1–21 days			
ADFI (g)	57.45 ± 2.34	57.00 ± 1.79	0.515
ADG (g)	48.87 ± 1.91	50.08 ± 1.46	<0.05
FCR	1.18 ± 0.02	1.14 ± 0.03	<0.001
22–42 days			
ADFI (g)	147.73 ± 5.41	148.11 ± 3.74	0.826
ADG (g)	83.28 ± 3.84	92.02 ± 4.75	<0.001
FCR	1.72 ± 0.09	1.60 ± 0.06	<0.001
1–42 days			
ADFI (g)	101.97 ± 2.90	102.75 ± 2.08	0.378
ADG (g)	66.20 ± 2.07	71.03 ± 2.16	<0.001
FCR	1.54 ± 0.03	1.46 ± 0.04	<0.001
1-day BW (g)	45.31 ± 1.02	45.05 ± 1.25	0.617
21-day BW (g)	1071.684 ± 40.17	1096.78 ± 30.62	<0.05
42-day BW (g)	2825.77 ± 86.86	3029.23 ± 103.53	<0.001

Values are expressed as means ± SD; CN = control group; BS = *Brevibacillus laterosporus* S62-9 group; ADFI = average daily feed intake; ADG = average daily gain; FCR = feed conversion ratio; BW = body weight; *p* < 0.05 was taken to indicate statistical significance.

**Table 3:** Effects of dietary *Brevibacillus laterosporus* S62-9 on the carcass traits of chicken meat.

Item	CN	BS	*p*-Value
Dressing percentage (%)	94.10 ± 1.57	94.49 ± 0.98	0.447
Semi-eviscerated percentage (%)	83.76 ± 2.92	84.46 ± 2.50	0.484
Eviscerated percentage (%)	74.48 ± 2.69	75.71 ± 2.60	0.206
Abdominal fat (%)	1.21 ± 0.27	1.20 ± 0.19	0.890
Breast muscle (%)	28.07 ± 1.58	30.82 ± 1.33	<0.001
Thigh muscle (%)	19.47 ± 1.16	18.85 ± 1.27	0.289

Values are expressed as means ± SD; CN = control group; BS = *Brevibacillus laterosporus* S62-9 group; *p* < 0.05 was taken to indicate statistical significance.

**Table 4:** Effects of dietary *Brevibacillus laterosporus* S62-9 on the meat quality of chicken meat.

Item	Muscle	CN	BS	*p*-Value
45 min pH	Breast	6.52 ± 0.15	6.43 ± 0.08	0.149
	Thigh	6.69 ± 0.08	6.60 ± 0.04	<0.01
24 h pH	Breast	6.10 ± 0.11	6.17 ± 0.09	0.149
	Thigh	6.51 ± 0.13	6.29 ± 0.20	<0.05
24 h L*	Breast	53.15 ± 2.62	50.54 ± 2.15	<0.05
	Thigh	51.82 ± 2.08	53.08 ± 2.95	0.283
24 h a*	Breast	4.67 ± 1.74	7.21 ± 2.27	<0.05
	Thigh	6.67 ± 2.92	6.04 ± 2.18	0.632
24 h b*	Breast	9.55 ± 1.43	9.64 ± 1.39	0.888
	Thigh	8.75 ± 1.72	9.65 ± 0.86	0.153
Shear force (N)	Breast	27.23 ± 6.09	22.99 ± 6.18	<0.05
	Thigh	20.99 ± 3.04	17.71 ± 3.43	<0.01
Pressing loss (%)	Breast	17.56 ± 7.72	17.19 ± 2.90	0.889
	Thigh	14.67 ± 2.69	10.53 ± 3.08	<0.05
Cooking loss (%)	Breast	16.14 ± 2.61	14.11 ± 3.68	0.225
	Thigh	14.59 ± 0.35	11.60 ± 2.30	<0.05

Values are expressed as means ± SD; CN = control group; BS = *Brevibacillus laterosporus* S62-9 group; L*, lightness; a*, redness; b*, yellowness; *p* < 0.05 was taken to indicate statistical significance.

**Table 5:** Effects of dietary *Brevibacillus laterosporus* S62-9 on the chemical composition of chicken meat.

Item	Muscle	CN	BS	*p*-Value
Moisture (%)	Breast	72.38 ± 0.60	72.57 ± 1.70	0.869
	Thigh	71.68 ± 1.56	70.30 ± 2.09	0.410
Protein (%)	Breast	19.81 ± 0.59	20.83 ± 0.67	<0.05
	Thigh	19.35 ± 0.72	19.21 ± 0.21	0.650
Fat (%)	Breast	2.88 ± 0.21	3.12 ± 0.35	0.378
	Thigh	5.60 ± 1.16	8.15 ± 0.61	<0.05
ash (%)	Breast	1.23 ± 0.12	1.13 ± 0.12	0.349
	Thigh	1.02 ± 0.76	1.01 ± 0.93	0.892

Values are expressed as means ± SD; CN = control group; BS = *Brevibacillus laterosporus* S62-9 group; *p* < 0.05 was taken to indicate statistical significance.

**Table 6:** Effects of dietary *Brevibacillus laterosporus* S62-9 on the amino acid content of chicken meat (g/100 g chicken meat).

Amino Acids	Breast			Thigh		
	CN	BS	*p*-Value	CN	BS	*p*-Value
Essential amino acid (EAA)						
Thr	0.72 ± 0.03	0.81 ± 0.05	<0.05	0.59 ± 0.06	0.63 ± 0.05	0.332
Val	0.85 ± 0.04	0.93 ± 0.06	0.077	0.65 ± 0.07	0.69 ± 0.04	0.352
Met	0.46 ± 0.02	0.52 ± 0.03	<0.05	0.35 ± 0.07	0.38 ± 0.03	0.414
Ile	0.84 ± 0.04	0.91 ± 0.05	0.172	0.64 ± 0.08	0.66 ± 0.04	0.516
Leu	1.49 ± 0.07	1.59 ± 0.08	0.340	1.14 ± 0.16	1.18 ± 0.08	0.521
Phe	0.66 ± 0.03	0.73 ± 0.04	<0.05	0.53 ± 0.06	0.55 ± 0.03	0.413
Lys	1.57 ± 0.07	1.7 ± 0.07	0.138	1.23 ± 0.15	1.28 ± 0.08	0.472
Non-essential amino acid (NEAA)						
Asp	1.56 ± 0.07	1.72 ± 0.09	0.051	1.24 ± 0.14	1.30 ± 0.09	0.403
Ser	0.62 ± 0.03	0.69 ± 0.04	<0.05	0.53 ± 0.06	0.57 ± 0.04	0.265
Glu	2.65 ± 0.12	2.83 ± 0.14	0.229	2.19 ± 0.29	2.33 ± 0.16	0.320
Gly	0.72 ± 0.04	0.84 ± 0.06	<0.05	0.65 ± 0.08	0.66 ± 0.03	0.686
Ala	0.96 ± 0.04	1.09 ± 0.06	<0.05	0.79 ± 0.08	0.82 ± 0.05	0.404
Cys	0.03 ± 0.01	0.04 ± 0.01	0.298	0.05 ± 0.01	0.04 ± 0.01	0.364
Tyr	0.54 ± 0.02	0.53 ± 0.16	0.697	0.45 ± 0.05	0.47 ± 0.04	0.475
His	0.65 ± 0.05	0.70 ± 0.05	0.306	0.40 ± 0.05	0.42 ± 0.04	0.397
Arg	1.09 ± 0.05	1.24 ± 0.07	<0.05	0.89 ± 0.09	0.94 ± 0.06	0.380
Pro	0.55 ± 0.03	0.57 ± 0.06	0.980	0.49 ± 0.07	0.51 ± 0.03	0.656
Total EAA	6.75 ± 0.47	7.18 ± 0.35	0.104	5.13 ± 0.64	5.37 ± 0.34	0.436
Total AA	15.96 ± 0.73	17.45 ± 0.85	0.106	12.81 ± 1.50	13.42 ± 0.81	0.400

Values are expressed as means ± SD; CN = control group; BS = *Brevibacillus laterosporus* S62-9 group; total EAA, the sum of Thr, Val, Met, Ile, Leu, Phe, and Lys; total AA, the sum of all detected amino acids; *p* < 0.05 was taken to indicate statistical significance.

**Table 7:** Effects of dietary *Brevibacillus laterosporus* S62-9 on the volatile compounds of chicken meat (μg/kg chicken meat).

NO.	Compounds	Odor Description	Aroma Intensity					
			Breast			Thigh		
			CN	BS	*p*-Value	CN	BS	*p*-Value
	Aldehydes							
1	2-methyl-butanal	Musty, chocolate, nutty	8.51 ± 4.89	22.07 ± 9.20	<0.001	12.54 ± 1.51	8.25 ± 6.47	0.145
2	Hexanal	Green, grassy	29.72 ± 20.15	593.71 ± 144.54	<0.001	9.08 ± 4.18	170.22 ± 176.56	<0.05
3	Octanal	Fruity, sweet orange	7.68 ± 4.59	63.75 ± 36.94	<0.01	5.35 ± 1.56	10.64 ± 9.99	0.228
4	Nonanal	Sweet melon	54.23 ± 46.27	125.33 ± 75.4	0.077	24.36 ± 11.09	18.84 ± 8.85	0.364
5	(E)-2-heptenal	Medicinal	17.10 ± 6.26	37.47 ± 26.97	0.102	-	9.22 ± 10.58	-
6	(E)-2-octenal	Grilled meat, peanut cake	18.23 ± 6.26	39.29 ± 24.43	0.068	-	-	-
7	(E)-2-decenal	Roasted, nut, crust	9.85 ± 2.78	11.41 ± 5.73	0.560	2.25 ± 0.43	-	-
8	(E)-2-undecenal	Fresh fragrance, cabbage	6.86 ± 2.86	5.32 ± 3.84	0.452	-	-	-
9	Heptanal	Fresh, aldehydic, fatty	-	43.39 ± 16.74	-	-	17.08 ± 14.81	-
10	Decanal	Green	-	6.49 ± 2.2	-	-	-	-
11	(E,E)-2,4-nonadienal	Toasted, fatty	-	5.44 ± 3.29	-	-	-	-
12	(E,E)-2,4-decadienal	Fatty, toasted, scallion	13.80 ± 2.73	3.63 ± 2.80	<0.001	8.82 ± 1.14	-	-
	Alcohols							
13	1-Pentanol	Pungent, fermented	24.59 ± 3.14	75.35 ± 44.26	<0.05	29.45 ± 4.65	42.57 ± 25.88	0.250
14	(E)-2-octen-1-ol	Green, citrus, vegetable	45.2 ± 37.55	42.62 ± 9.57	0.874	15.47 ± 3.25	14.30 ± 7.65	0.739
15	1-octanol	Waxy, green, orange	45.81 ± 25.15	62.03 ± 24.59	0.285	26.72 ± 7.22	7.86 ± 4.59	<0.001
16	4-ethylcyclohexanol	-	44.45 ± 24.99	37.13 ± 26.19	0.631	12.91 ± 1.79	-	-
17	1-nonanol	Fresh, clean, fatty	107.19 ± 36.90	-	-	37.80 ± 8.83	-	-
18	1-octen-3-ol	Mushroom	127.75 ± 32.17	331.66 ± 136.34	<0.01	87.31 ± 15.63	54.03 ± 59.28	0.213
19	2-ethyl-1-hexanol	Citrus, fresh, floral	6.53 ± 1.27	5.72 ± 1.79	0.383	-	6.52 ± 1.84	-
20	3-(methylthio)-1-propanol	Sulfurous, onion, sweet	14.60 ± 0.01	-	-	6.47 ± 1.67	-	-
21	1-hexanol	Ethereal, fusel, oily	292.53 ± 146.01	285.61 ± 161.76	0.939	169.87 ± 49.31	-	-
22	1-heptanol	Musty, leafy, violet	-	54.66 ± 15.5	-	20.8 ± 4.83	7.99 ± 9.50	<0.05
23	1-nonen-4-ol	-	-	15.93 ± 1.54	-	4.93 ± 0.63	-	-
	Acid							
24	Propanoic acid	Pungent, acidic	14.93 ± 200.20	-	-	5.47 ± 4.82	-	-
25	Octanoic acid	Fatty, waxy, rancid	14.13 ± 5.89	3.79 ± 1.16	<0.01	6.55 ± 3.7	1.22 ± 0.43	<0.01
26	Nonanoic acid	Waxy, dirty, cheese	2.40 ± 1.74	1.80 ± 0.01	0.414	1.64 ± 0.84	4.48 ± 2.97	<0.05
	Others							
27	2-pentylfuran	Fruity, green, earthy	36.03 ± 21.02	28.17 ± 21.94	0.540	9.48 ± 3.05	4.48 ± 1.37	<0.01
28	2-nonanone	Fresh, sweet, green	22.11 ± 15.02	14.54 ± 19.99	0.539	8.50 ± 3.87	-	-
29	(E,E)-3,5-octadien-2-one	Fruity, green, grassy	6.61 ± 0.61	4.80 ± 1.45	<0.05	-	-	-
30	2,3-octanedione	Dill, asparagus	-	47.27 ± 8.48	-	-	17.44 ± 19.27	-
31	p-cresol	Phenolic, narcissus, animal	-	0.40 ± 0.05	-	0.92 ± 0.31	0.69 ± 0.21	0.157

Values are expressed as means ± SD; CN = control group; BS = *Brevibacillus laterosporus* S62-9 group; Compounds, divided into four categories according to the functional group; *p* < 0.05 was taken to indicate statistical significance.

**Table 8:** The ROAVs of the volatile compounds of chicken meat.

NO.	Compounds	Thresholds (μg/kg)	ROAVs					
			Breast			Thigh		
			CN	BS	*p*-Value	CN	BS	*p*-Value
	Aldehydes							
1	2-methyl-butanal	84.300	<1	<1	-	<1	<1	0.145
2	Hexanal	5.000	6	119	<0.001	2	34	<0.05
3	Octanal	0.587	13	109	<0.01	9	18	0.228
4	Nonanal	1.100	49	114	0.077	22	17	0.364
5	(E)-2-heptenal	40.000	<1	<1	-	-	-	-
6	(E)-2-octenal	3.000	6	13	0.068	-	-	-
7	(E)-2-decenal	17.000	<1	<1	-	<1	-	-
8	(E)-2-undecenal	0.780	9	7	0.452	-	-	-
9	Heptanal	2.800	-	15	-	-	-	-
10	Decanal	3.000	-	2	-	-	-	-
11	(E,E)-2,4-nonadienal	0.100	-	54	-	-	-	-
12	(E,E)-2,4-decadienal	2150.000	<1	<1	-	<1	-	-
	Alcohols							
13	1-pentanol	150.200	<1	<1	-	<1	<1	-
14	(E)-2-octen-1-ol	20.000	2	2	0.874	<1	<1	-
15	1-octanol	125.800	<1	<1	-	<1	<1	-
16	4-ethylcyclohexanol	-	-	-	-	-	-	-
17	1-nonanol	45.500	2	-	-	<1	-	-
18	1-octen-3-ol	1.500	85	221	<0.01	58	36	0.213
19	2-ethyl-1-hexanol	25482.200	<1	<1	-	-	<1	-
20	3-(methylthio)-1-propanol	123.230	<1	-	-	-	-	-
21	1-hexanol	500.000	<1	<1	-	-	-	-
22	1-heptanol	425.000	-	<1	-	<1	<1	-
23	1-nonen-4-ol	-	-	-	-	-	-	-
	Acid							
24	Propanoic acid	2190.000	<1	-	-	<1	-	-
25	Octanoic acid	3000.000	<1	<1	-	<1	<1	-
26	Nonanoic acid	4600.000	<1	<1	-	<1	<1	-
	Others							
27	2-pentylfuran	5.800	6	5	0.540	2	<1	<0.01
28	2-nonanone	41.000	<1	<1	-	<1	-	-
29	(E,E)-3,5-octadien-2-one	100.000	<1	<1	-	-	-	-
30	2,3-octanedione	-	-	-	-	-	-	-
31	p-cresol	3.900	-	<1	-	<1	<1	-

CN = control group; BS = *Brevibacillus laterosporus* S62-9 group; NO.: consistent with the compound number in Table 7; threshold values in water were expressed; “<1” was used for ROAV values less than 1; *p* < 0.05 was taken to indicate statistical significance.

**Table 9:** Effects of dietary *Brevibacillus laterosporus* S62-9 on the sensory properties of chicken meat.

Item	Breast			Thigh		
	CN	BS	*p*-Value	CN	BS	*p*-Value
Meat color	6.87 ± 1.20	7.11 ± 1.31	0.222	6.08 ± 1.41	6.23 ± 1.48	0.433
Aroma	6.70 ± 1.16	7.03 ± 1.06	0.187	6.24 ± 1.30	6.73 ± 1.11	<0.01
Springiness	6.13 ± 1.14	6.39 ± 1.11	0.178	6.02 ± 1.28	6.33 ± 1.45	0.097
Tenderness	6.07 ± 1.32	6.61 ± 1.29	<0.05	6.29 ± 1.45	6.73 ± 1.23	<0.05
Juiciness	5.83 ± 1.27	6.37 ± 1.32	<0.05	6.03 ± 1.36	6.16 ± 1.55	0.575
Taste	6.27 ± 1.17	6.38 ± 1.13	0.705	6.27 ± 1.24	6.47 ± 1.33	0.300
Umami	6.33 ± 1.18	6.29 ± 1.19	0.872	6.06 ± 1.58	6.20 ± 1.57	0.458
Aftertaste	6.39 ± 1.27	6.36 ± 1.13	0.751	6.18 ± 1.48	6.26 ± 1.65	0.739

Values are expressed as means ± SD; CN = control group; BS = *Brevibacillus laterosporus* S62-9 group; *p* < 0.05 was taken to indicate statistical significance.

## Data Availability

The datasets generated for this study are available on request to the corresponding author.

## Supplementary Materials

The following supporting information can be downloaded at: https://www.mdpi.com/article/10.3390/foods12020288/s1, Figure S1: PCA analysis of breast meat (A) and thigh meat (B), Table S1: Sensory attributes, definitions, and scales used to evaluate samples.
