# Supplementary material for: Dietary Effect of Brevibacillus laterosporus S62-9 on Chicken Meat Quality, Amino Acid Profile, and Volatile Compounds
Source: Foods. 2023 Jan 8;12(2):288. doi: 10.3390/foods12020288 (PMC9858446; doi:10.3390/foods12020288)
Supplement: Supplementary file 1 [file foods-12-00288-s001.zip › foods-2081801-supplementary.pdf]

Table S1. Sensory attributes, definitions, and scales used to evaluate samples.

Attribute	Definition	Scale
Meat color	The intensity of light and dark of the cooked meat.	disliked very much- liked very much
Aroma	The intensity of the odor that can be detected.	
Springiness	The ability to recover immediately from depressions after fingering	
Tenderness	Force required to chew the piece until it can be swallowed.	
Juiciness	Amount of fluid released during the first three chews.	
Taste	The intensity of all the flavors that can be felt during chewing.	
Umami	The intensity of umami taste.	
Aftertaste	The intensity of the aftertaste.	

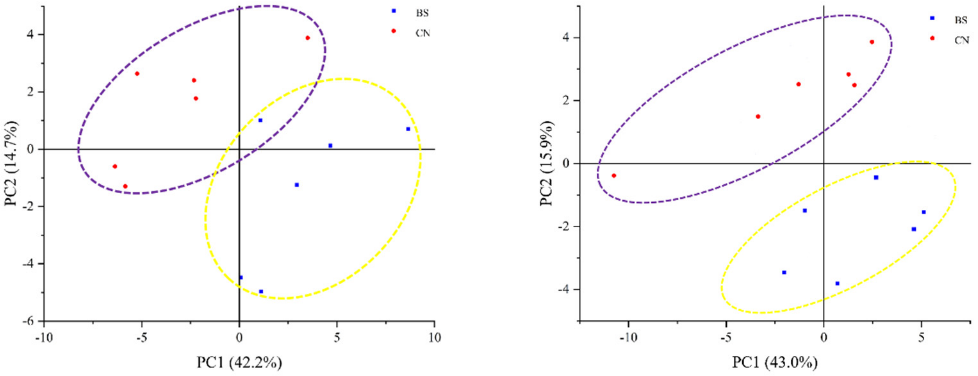

(A) **(B)**

Figure S1. PCA analysis of breast meat (A) and thigh meat (B). Red squares represent the control group and blue squares represent the experimental group.
